# BONCAT-Live for isolation and cultivation of active environmental bacteria

**DOI:** 10.1128/mbio.02389-25

**Published:** 2025-09-22

**Authors:** Sayali A. Mulay, Tatiana A. Vishnivetskaya, Leah H. Hochanadel, Dawn M. Klingeman, Karen G. Lloyd, Dale A. Pelletier, Mircea Podar

**Affiliations:** 1 Biosciences Division, Oak Ridge National Laboratory 6146, Oak Ridge, Tennessee, USA; 2 Department of Microbiology, University of Tennessee Knoxville 4292, Knoxville, Tennessee, USA; 3 USC Dana and David Dornsife College of Letters, Arts and Sciences, University of Southern California 5116, Los Angeles, California, USA; The University of Oklahoma, Norman, Oklahoma, USA

**Keywords:** microbial cultivation, rhizosphere-inhabiting microbes, human microbiome, permafrost, BONCAT

## Abstract

In diverse environments, microbes drive a myriad of processes, from geochemical and nutrient cycling to interspecies interactions, including associations with plants and animals. Their physiological state is dynamic and impacted by abiotic and biotic conditions, responding to environmental fluctuations by changes in cellular metabolism, according to their genetic potential. Molecular, cellular, and genomic approaches can identify and measure microbial responses and adaptation to environmental changes in native communities. However, isolating individual microbial cells that respond to specific changes for cultivation has been difficult. To address this, we implemented a novel bacterial isolation approach (BONCAT-Live) by integrating bio-orthogonal non-canonical amino acid tagging (BONCAT) in diverse native communities, with isolation and cultivation of cells responding to specific stimuli, at different time scales. In frozen Arctic permafrost samples, we identified and isolated dormant bacteria that become active after thawing under native or nutrient-enriched conditions. From the *Populus* tree rhizosphere, we isolated strains that thrive under high concentrations of root exudates that act as defense compounds and nutrients. In the human microbiome, we identified and isolated bacteria that rapidly proliferated when exposed to metabolites provided by the host or other co-occurring microbes. Further characterization of isolated bacterial strains will provide opportunities for in-depth determination of how these microbes adapt to changes in their environments, individually and as part of model communities.

**IMPORTANCE:**

Dynamic microbial activity transforms environments and impacts health and disease in associations with plants and animals, including humans. Identifying the contribution of individual microbes to those processes in real time has not been generally compatible with their selective cultivation. BONCAT-Live tracks which bacteria in environmental samples are translationally active and couples it with single-cell isolation and cultivation. By studying the response of individual community members to specific natural or induced physical or chemical changes in the environment and culturing those organisms, BONCAT-Live enables further insights into microbial metabolic strategies, community dynamics, and environmental adaptations.

## INTRODUCTION

Most environments harbor complex microbial communities, taxonomically diverse, partitioned across various niches and ranging from specialists to physiologically versatile. Fluctuations of physical, chemical, and biotic parameters, at different spatial and temporal scales, can selectively impact and partition the metabolic states of individual members of the community. Taxa that are under permissive conditions (temperature, nutrients, energy sources, etc.) would display various levels of physiological activity (e.g., respiration, protein synthesis, and cell division), while those facing adversities and limitations may enter dormancy (including sporulation and viable but non-culturable state) (1–3) until optimal conditions resume. Physiological activation from dormant states (as evidenced by gene expression, protein synthesis, cell division, motility, etc.) can occur rapidly, over minutes or hours, even after years (4, 5). In some microbes, metabolic activity can persist across a wide range of conditions (e.g., low temperature-adapted microbes) by adopting distinct strategies (genes and metabolites) at different states (6–8). When membrane integrity is compromised (cell death), DNA can persist and is detectable for some time, which has prompted the development of specific approaches for distinguishing such microbial relics from viable and active cells (9). Distinguishing live and dead cells is, however, not always straightforward, as the degree of membrane polarization can mislead assay interpretation for some organisms (10). At the community level, a wide range of techniques can detect and quantify specific biochemical processes (e.g., respiration, denitrification, methanogenesis, and sulfate reduction) *in situ* and in the laboratory. By using stable isotope probing (SIP) coupled with metagenomic sequencing, physiologically active microbes can be identified and quantified (11) and, when combined with spectroscopic techniques, dissected at cellular and subcellular levels (12, 13). A variety of metabolic substrate analogs have been developed to track specific cellular processes and how microbes react under changing conditions (12). Cell surface labeling using click chemistry has been demonstrated by using analogs incorporated into the peptidoglycan and the outer membrane (14–16). However, because of the many differences in the structure and cellular envelope synthesis across microbes, there is no single, broadly applicable substrate for environmental tagging. Bio-orthogonal non-canonical amino acid tagging (BONCAT) uses amino acid analogs that can be conjugated via click reactions to chemical tags (e.g., fluorophores), to label proteins in translationally active cells (17, 18), including in environmental samples (19–24). Those cells can subsequently be identified and visualized via microscopy-fluorescence *in situ* hybridization (FISH) or isolated by fluorescence-activated cell sorting (FACS) (BONCAT-FACS) followed by amplicon sequencing (22, 23). So far, and to our knowledge, BONCAT has only been applied in cultivation-independent studies, without recovering viable microbes for subsequent research.

Microbial isolation and cultivation, whether by traditional or high-throughput approaches, rely on using a broad range of conditions, from highly selective to broadly permissive in nutrients, energy sources, and temperature (e.g., see references 25–31). This leads to isolates that may or may not have been highly active in the environment at the time of collection or indicative of their responses to changing conditions or interactions with other species. Here, we have implemented BONCAT-Live as an approach to isolate and culture translationally active bacteria that respond to simulated environmental changes at different time scales, from weeks to hours. Specifically, we modeled permafrost thawing, the responses of *Populus* tree rhizosphere to plant-secreted metabolites and that of human oral microbiota to host and community shared nutrients.

## RESULTS

### Cell surface click-chemistry and viability tests enable BONCAT-Live

**Fig 1:**
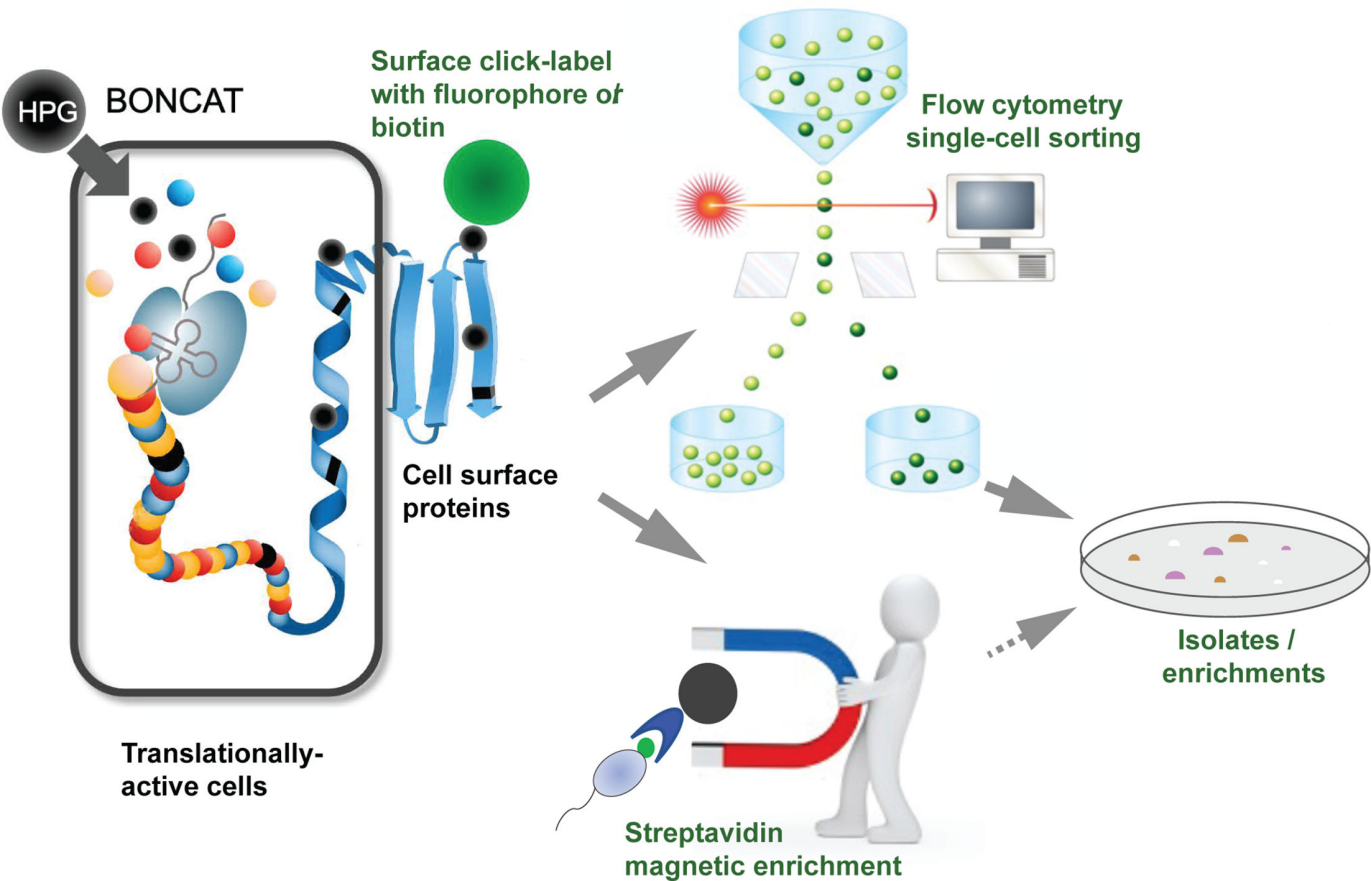
Concept and workflow diagram for BONCAT-Live.

**Fig 2:**
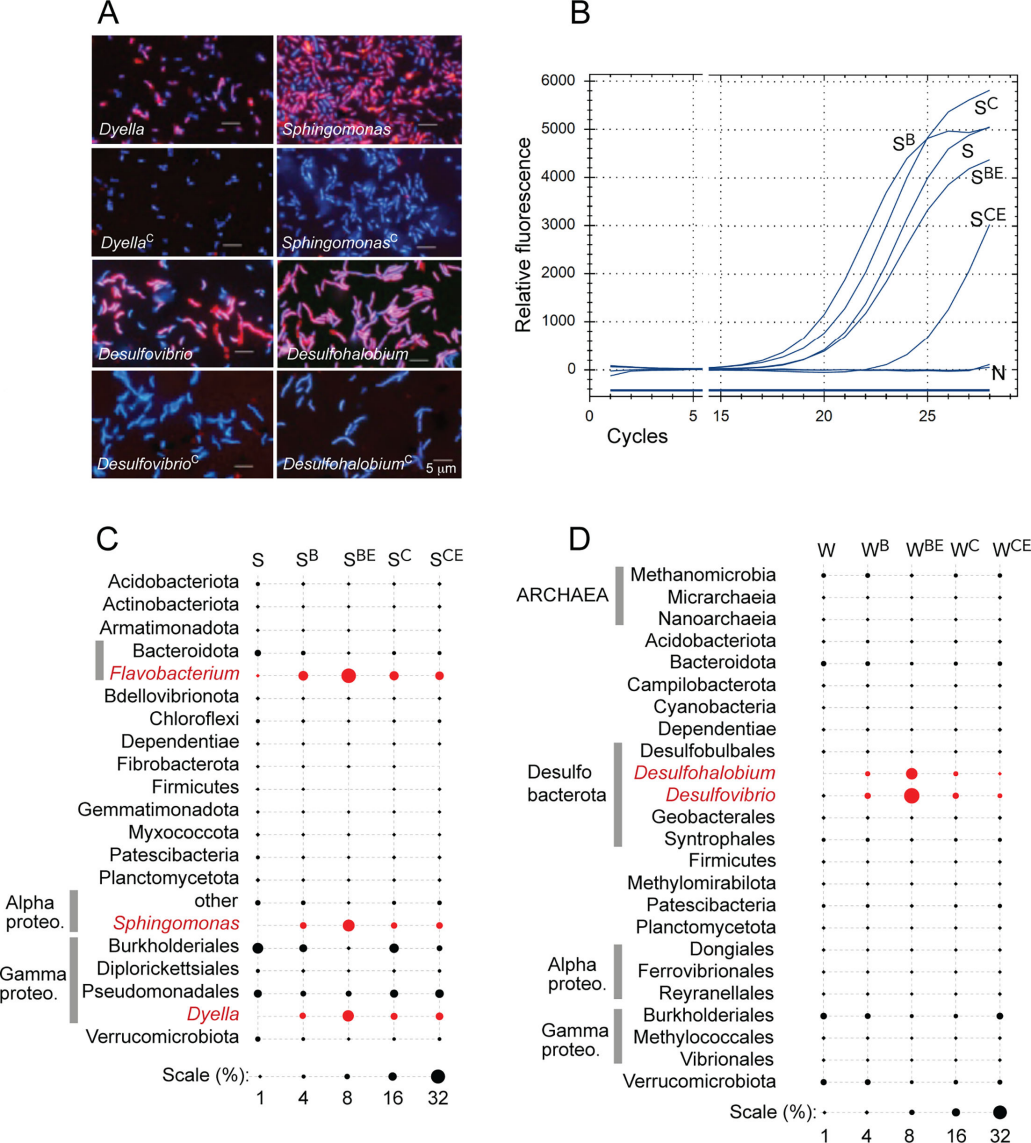
Cell surface BONCAT labeling and selection. (**A**) Strains of five bacterial genera grown in the presence or absence of HPG (controls, superscript C) were labeled with biotin and imaged after surface staining with Streptavidin-A546. Aliquots of those cells were also mixed in a soil (S) or freshwater sediment (W) microbiota sample, labeled with biotin (superscript B) by clickchemistry and magnetically enriched (superscript E). The bubble plots (**C, D**) indicate taxon relative abundance (in %) based on SSU rRNA V4 amplicons comparing the samples before and after seeding and after elution from the magnetic column. The real-time PCR plot (**B**) is shown for the amplification of the soil samples (N is the negative control).

Previous studies that applied BONCAT to characterize active microbial populations have primarily focused on labeling and detection efficiency. Because some of the reagents used in the click-chemistry reactions may not effectively pass through the cellular membranes, fixation has been routinely used to enable access to cytoplasmic proteins, which are expected to be the major targets for tagging. Testing whether surface-exposed proteins may be sufficient substrates for click-chemistry tagging of cells with an intact membrane was a supporting step in evaluating BONCAT-Live (Fig. 1), expanding on previously published experiments with live cells (17). In three independent experiments, we grew pure cultures of phylogenetically and physiologically diverse bacteria (*Terriglobus* sp., *Flavobacterium* sp., *Bacillus* sp., *Rhizobium* sp., *Paraburkholderia* sp. *Roseimicrobium* sp., *Sphingomonas* sp., *Dyella* sp., *Desulfovibrio desulfuricans*, and *Desulfohalobium retbaense*) (32) in the presence of *L*-homopropargylglycine (HPG) and performed fixation-independent copper (Cu)-mediated click reactions with a biotin-azide tag. Microscopic examination after labeling with fluorescent streptavidin revealed successful and specific tagging at membrane level (Fig. 2; Fig. S1). Notably, for some strains and based on DNA counterstaining, not all cells were labeled equally. This may be linked to cell death, dormancy, differential cell wall and membrane complex assembly, variables that are expected to occur in nature as well. As a macromolecule, streptavidin is not expected to pass through intact plasma membranes but may have different accessibility to proteins in the different types of bacterial cell walls. As a separate test to determine if the biotin tags are physically accessible on the cell surface, we also seeded those HPG-containing bacteria and controls into environmental microbiota samples (*Populus* rhizosphere, garden soil, and pond surface sediment). After the click reaction with biotin-azide, we used streptavidin-coated nanobeads to capture labeled cells using a magnetic separation column. Small subunit (SSU) rRNA gene V4 amplicon sequencing of the samples, before and after magnetic capture, shows that the seeded bacteria grown in the presence of HPG were preferentially enriched (typically two- to fourfold) as compared to non-HPG treated controls (Fig. 2; Fig. S1). The column matrix leads to some non-specific microbial trapping, which was evidenced by delayed amplification profiles and different taxonomic profiles between control and labeled populations. While not intended to be quantitative or lead to cell purification, these experiments demonstrate the accessibility of HPG-containing surface proteins to tagging for detection and isolation. The approach may be further optimizable to achieve higher enrichment (e.g., by performing multiple rounds of magnetic separation) with samples/organisms that would be difficult to process for FACS (e.g., due to cell size, sensitivity to oxygen, or mechanical stress).

Next, we investigated how the treatment steps and chemicals associated with BONCAT, click chemistry, and sorting (whether by using cell scattering properties or fluorescent labels, i.e., FACS) impact microbial viability, as each subjects the cells to different stresses. While we recognize that the many species of microbes present in the environment may respond differently, we evaluated those parameters primarily using a reference *Pseudomonas* sp. GM41 isolated from *Populus deltoides* rhizosphere (32). The assay end point was colony formation (counts) after sorting of 100 individual particles/cells onto R2A nutrient agar (Fig. S2). The addition of either azidohomoalanine (AHA, 50–100 μM) or HPG (50–100 μM) during culture incubation did not noticeably affect viability (>80%, Fig. S2), supporting prior reports of low toxicity in pure cultures and environmental samples at those concentrations (17, 33, 34). Bio-orthogonal amino acids can have different toxic effects on some organisms, depending on concentration and culture conditions (35). We also investigated the effect of click labeling reactions on viability. For strain-promoted, Cu-independent click chemistry labeling with a dibenzocyclooctyne-containing dye following AHA incorporation, free thiols on the cell surface need to be blocked first. That reaction requires a high concentration of 2-chloroacetamide (10–100 mM), a toxic compound, and resulted in poor cell viability (no growth at 100 mM, <10% at 10 mM; plate images not shown). We focused, therefore, on Cu-mediated click chemistry coupled with fluorescent AZDye 488 picolyl azide cellular labeling, following incubations with HPG only. The individual compounds necessary to generate the reduced environment and block non-specific reactions (ascorbate and aminoguanidine) were not significantly toxic at the standard concentrations (up to 5 mM) and incubation times (30–60 min) for click-labeling reactions. The typically used Cu concentration (50 µM) had a more detrimental impact on viability, with >50% inhibition (Fig. S2). While an essential microelement, Cu can be toxic by generating reactive oxygen species (ROS), inactivating iron-sulfur clusters in essential enzymes, and microbes have evolved various mechanisms to counteract it (36, 37). After multiple tests to mitigate Cu toxicity during click reactions, we found that reducing its concentration to 5 µM and including ROS scavengers (pyruvate and catalase) maintained efficient click labeling while improving viability to ~80%, conditions we further applied to the BONCAT-Live studies. Pyruvate and catalase have been previously found to improve microbial viability and cultivability (38–40) and may be especially beneficial for organisms that are sensitive to ROS.

### Microbes in the *Populus* rhizosphere are stimulated by root exudates

Plants establish symbiotic associations with microbes and fungi in the rhizosphere and within the roots by producing diverse chemical cues that reshape the diversity relative to the surrounding soil (41–44). Among the compounds that plant roots exude at various levels are amino acids, sugars, and organic acids which are used as nutrients by the surrounding microbiota. In addition, plants produce specific defense compounds to counteract pathogens and grazers. We used freshly collected *Populus deltoides* fine roots (2–3 mm thick) with the immediately surrounding soil. Root exudates often form a concentration gradient that is dependent on the amount and rate of secretion, soil diffusion, and rate of utilization by different microorganisms. Some compounds, including antimicrobial secondary metabolites, can be utilized sequentially by distinct microbial guilds and provide nutritional handoffs (cross-feeding) to other taxa (44, 45). Also, under native environmental conditions limited by water, nutrient availability, and competition/predation, microbes have variable effective growth/division rates, ranging from hours to days (46). Based on preliminary tests, we detected BONCAT-positive rhizosphere bacteria within 12–48 hours of incubation. Within the same time frame, prior studies have applied BONCAT to identify active microbes under native or stimulated conditions of various soils (22, 47).

**Fig 3:**
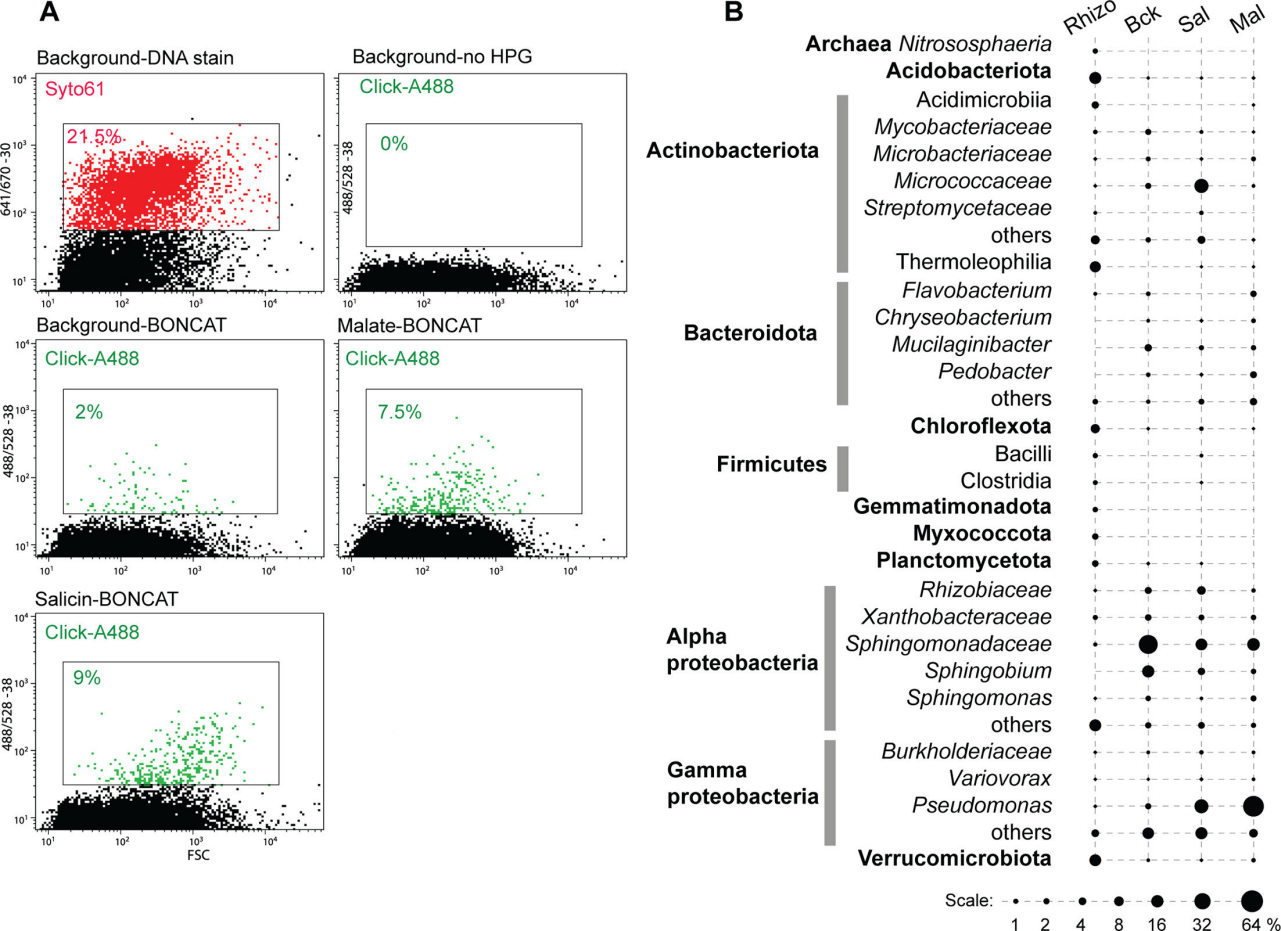
Rhizosphere BONCAT-Live experiment. (**A**) *Populus deltoides* rhizosphere samples were incubated for BONCAT with no exogenous nutrients (background), with salicin (Sal) or malate (Mal), followed by click labeling with Alexa Fluor 488, DNA staining (Syto61), and flow cytometry cell sorting. Cytometry plots and color gates indicate frequency of labeled particles for control and BONCAT-labeled samples (DNA staining not shown for all samples). (**B**) Microbial taxa relative abundance (%) in BONCAT-positive sorted cell populations based on gating shown in panel **A**.

**Fig 4:**
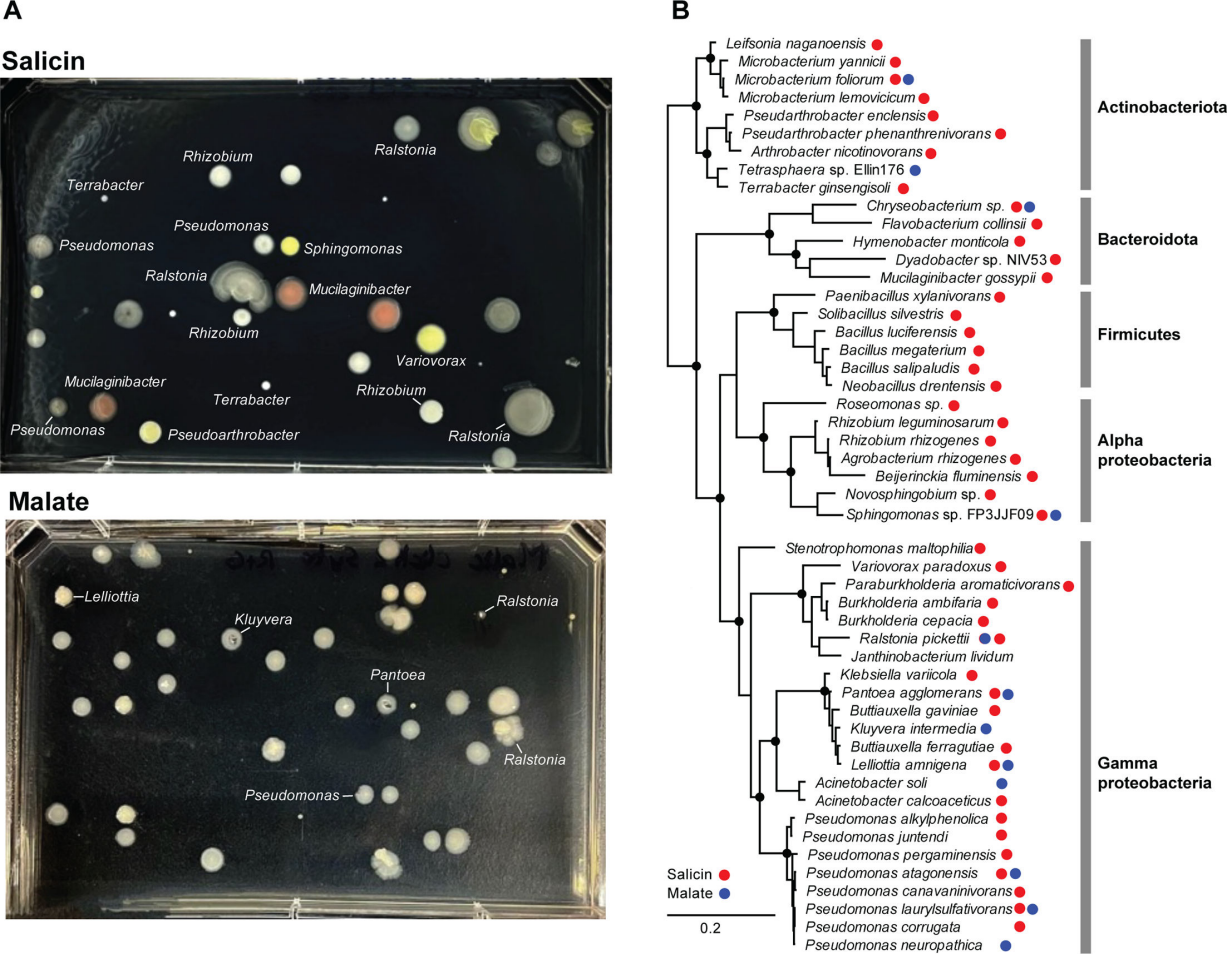
Microbial isolates from rhizosphere BONCAT-Live. (**A**) Single cells from salicin and malate gates were sorted on R2A agar and incubated to form colonies. Shown are example plates and identity of selected bacterial colonies based on SSU rRNA amplicon sequencing. (**B**) SSU rRNA phylogeny of bacterial species for which strains were isolated from salicin and malate BONCAT-Live.

The *Populus* rhizosphere samples presented a rich microbial diversity at all taxa levels (>30 different phyla and >500 genera/species) (Fig. 3; Table S1). Background control incubations (fixed sample or without HPG) resulted in no label being incorporated by click chemistry, as determined by FACS. Incubations in the presence of HPG for 24 hours but with no supplemented growth substrates revealed a translationally active population of microbes (~2% of the DNA-positive particles) relying on soil and root-derived nutrients. Based on rRNA amplicon sequencing of labeled and sorted cells, alpha- and gamma-proteobacteria dominated, with members of the *Sphingomonadaceae* having the highest relative abundance. Based on this background level of activity, we supplemented the incubations with malate, a root-produced organic acid known to promote recruitment of beneficial bacteria (48). Following 24 hours, ~7.5% of the cells were BONCAT positive, dominated by *Pseudomonas* strains, as determined by amplicon sequencing (Fig. 3). From this population of translationally active cells, we performed FACS on single cells on R2A nutrient agar plates and incubated them to form colonies (Fig. 4). Based on full-length 16S rRNA gene amplification and sequencing, the isolates were assigned to *Pseudomonas*, other proteobacteria (*Pantoea*, *Ralstonia*, and *Sphingomonas*), as well as several members of Actinobacteriota and Bacteroidota (Fig. 4) known to associate with plant roots, including *Populus* (32).

From among the known *Populus* defense/regulatory exudates (49), we selected salicin, representing the salicylic acid plant hormonal and defense pathway (50, 51). Salicin is an abundant phenolic glycoside in *Populus*, and through stepwise enzymatic breakdown and cross-feeding among different bacteria (e.g., *Rahnella* and *Pseudomonas*), it has been proposed to contribute to the root microbiota assembly (45). Incubation of the rhizosphere-root samples with salicin resulted in 9% BONCAT-positive cells (Fig. 3). The highest increase in relative abundance, based on amplicon sequencing, was among Actinobacteriota (especially the *Micrococcaceae*), as well as *Pseudomonas* and other proteobacteria. Sorting of single cells on non-selective R2A agar media yielded two dozen distinct strains representing a rich diversity of Actinobacteriota, Bacteroidota, Firmicutes, and proteobacteria, close to many known root symbionts (Fig. 4). The incubations and sorting were performed several times, weeks apart with independent rhizosphere samples, and the isolates were analyzed collectively. We did not perform traditional cultivation in parallel with those samples as the open-ended nature of cultivation from high diversity microbiota samples would preclude significant correlations between isolates obtained using different approaches, at least at the depth we applied here.

### BONCAT-Live to study Arctic permafrost thawing

Permafrost is perennially frozen ground that covers a fifth of the Earth’s land surface (52) and stores half of the global soil organic carbon (53, 54). The permafrost lies beneath a surface soil layer that undergoes seasonal freeze-thaw cycles (active layer). As a result of global warming, the active layer that is deepening and thawing reaches into the permafrost. Microbes that have been frozen for up to millions of years can become metabolically active and break down organic matter, releasing greenhouse gases into the atmosphere and creating a positive feedback loop (55). As part of a broader study of permafrost communities, we analyzed cores collected from the Bayelva site at Ny Ålesund, Svalbard (Norway), a location that is experiencing more extreme climate changes than the rest of the Arctic (56). In 2021, the recorded active layer temperature at this site was >0°C, with a maximum temperature of ~8°C (57). While a variety of biogeochemical and metagenomic approaches have been used to study thawing permafrost and its microbiota (58, 59), BONCAT has not been reported so far, to our knowledge. Additionally, BONCAT-Live could enable direct access to microbial strains as they become physiologically active under thawing conditions.

**Fig 5:**
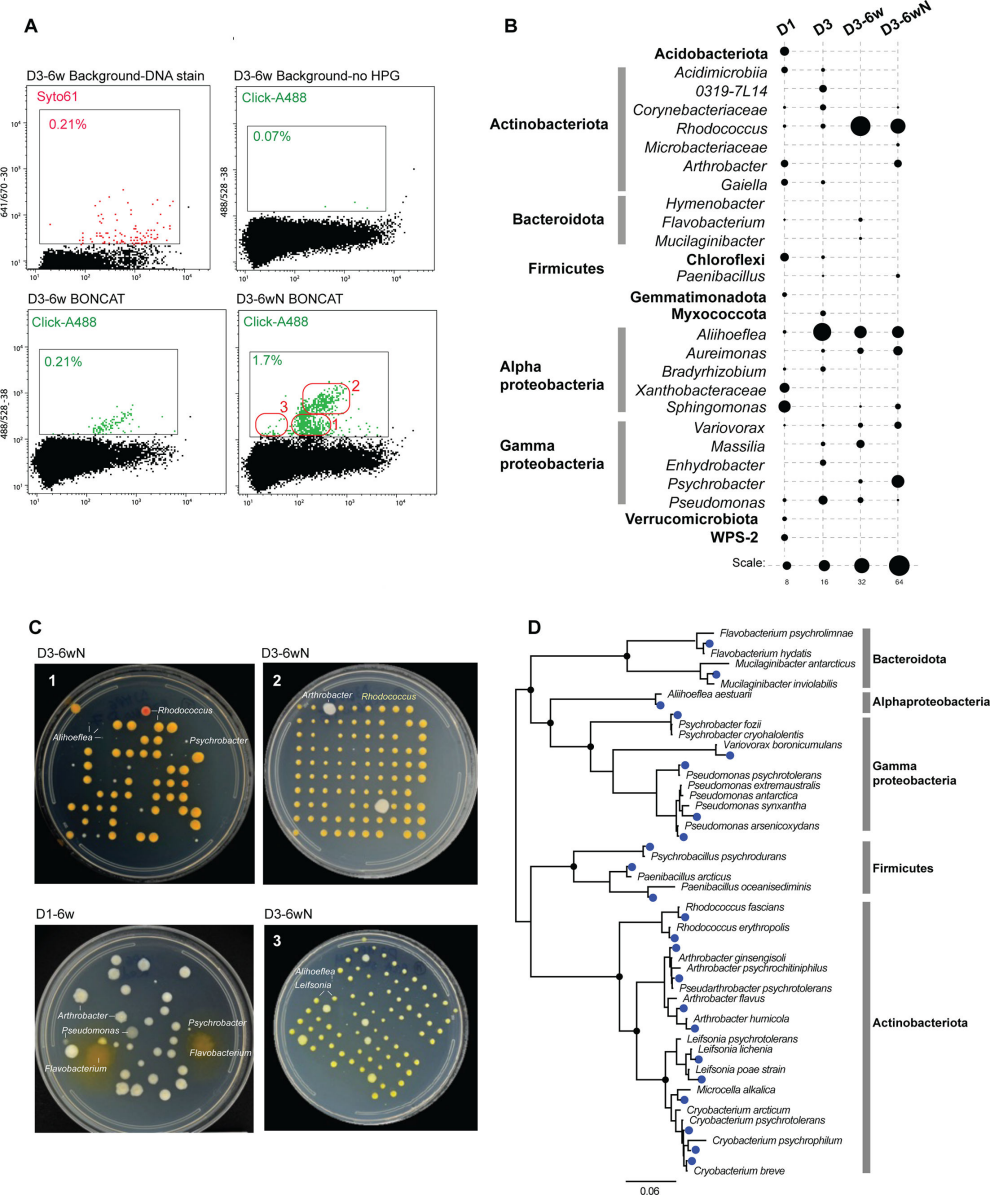
BONCAT-Live on Arctic permafrost cores. Samples from the active layer (D1) and permafrost (D3) were incubated for BONCAT without or with exogenous nutrients (N). (**A**) Flow cytometry plots for permafrost (D3) BONCAT, stained for DNA or click labeled with Alexa Fluor 488. Gates used for sorting and particle frequency are indicated. Nutrient-amended sample was fine gated (1–3) for bacterial isolation. (**B**) Microbial taxa relative abundance in original core samples (D1–D3) and BONCAT-positive sorted cell populations based on gating shown in panel **A**. (**C**) Bacterial isolates following BONCAT-positive single-cell sorting from non-amended and nutrient-enriched (N) permafrost incubations, based on gates shown in panel **A**. The identity of representative types of colonies is indicated. (**D**) SSU rRNA phylogeny of isolated bacterial strains (blue dots) and their closest relative described species.

Samples from frozen cores that spanned the active layer (about 0.5–0.7 m depth) and the deep permafrost (>2 m depth) had a distinct microbial composition. Some of the more typical soil taxa (including Acidobacteriota, Actinobacteriota, Gemmatimonadota, Chloroflexi, *Sphingomonas*, and Verrucomicrobiota) were represented in the active layers, while the permafrost had higher levels of various proteobacteria (*Aliihoeflea*, *Enhydrobacter*, and *Pseudomonas*) as well as Myxococcota and uncultured Actinobacteriota (Fig. 5). We tested the effects of incubation temperature (−20°C and 4°C) and time (2–6 weeks at 4°C and 12 weeks at −20°C) on BONCAT output (fluorescent particles following click labeling), cultivation temperature after cell sorting (15°C or 25°C), and media (standard/diluted nutrient agar). Because the site is characterized as a high-lithic and low-nutrient permafrost and permafrost-affected soils (60), we also supplemented some of the incubations with defined R2A-type nutrients.

The incubations performed at −20°C, as well as the negative BONCAT controls, yielded no click-labeling signal. The BONCAT-Live experiments were conducted mainly on 6 week incubations using permafrost layer samples and were performed twice, with subsamples from the same main core. During FACS analysis of the click-labeled samples, we sorted both entire labeled populations as well as subpopulations based on gates that suggested distinct scatter and fluorescence properties (Fig. 5). Based on amplicon sequencing of BONCAT-positive populations, specific taxa increased significantly in abundance relative to the starting permafrost. They included *Rhodococcus* and *Arthrobacter* (Actinobacteriota), *Aliihoeflea* and *Aureimonas* (Alphaproteobacteria), and *Psychrobacter* (Gammaproteobacteria). Supplementing the incubations with exogenous R2A-type nutrients had a relatively minor effect on alpha diversity, although nutrients stimulated some taxa and inhibited others, which was also evidenced in flow cytometry profiles (Fig. 5). Viability after single-cell deposition on media plates was comparable to what we had determined with tests on soil bacteria and with a *Pseudomonas* strain previously isolated from the same site (59). High viability was reflected in very high colony counts (up to ~90%) for some of cell populations sorted from the BONCAT permafrost incubations (Fig. 5). Colony morphology/color/size confirmed that some of the flow cytometry profiles/sorting gates were enriched in specific types of bacteria, some at very low abundance in the global community, identified following rRNA gene amplification and Sanger sequencing of representative colony morphotypes. Overall, using BONCAT-Live after two independent permafrost incubations performed months apart, we isolated approximately two dozen distinct strains representing five bacterial phyla and including potential novel species based on rRNA similarity to described species (Fig. 5). Several known psychrophiles (*Cryobacterium* and *Psychromonas*) were recovered primarily after plating at 15°C and displayed impaired growth at 25°C. Most of the other isolates grew faster at 25°C. Using standard versus diluted nutrient agar did not appear to influence the types of recovered strains, although we did not perform extensive comparisons.

### BONCAT-Live in oral microbiota

We also tested the applicability of BONCAT-Live in a more selective host-microbiota system, the human microbiome. Mammalian-associated microbes have adapted to specialized niches, and many rely on cross-feeding or host-supplied nutrients and cofactors. We selected the oral environment, as it encompasses a multitude of nutritional niches that harbor high taxonomic diversity and a combination of facultative and strict anaerobes. BONCAT has already been demonstrated as a feasible approach in studying oral microbiota (17). Here, we investigated several nutrients that support and modulate interspecies interactions in saliva and supragingival biofilm. Because many oral bacteria are anaerobes, we performed all incubations, click labeling, and FACS under anoxic conditions. In addition, due to the generally fast growth rate and to avoid extensive interspecies cross-feeding, we used a shorter incubation time than for environmental samples: 2 hours.

**Fig 6:**
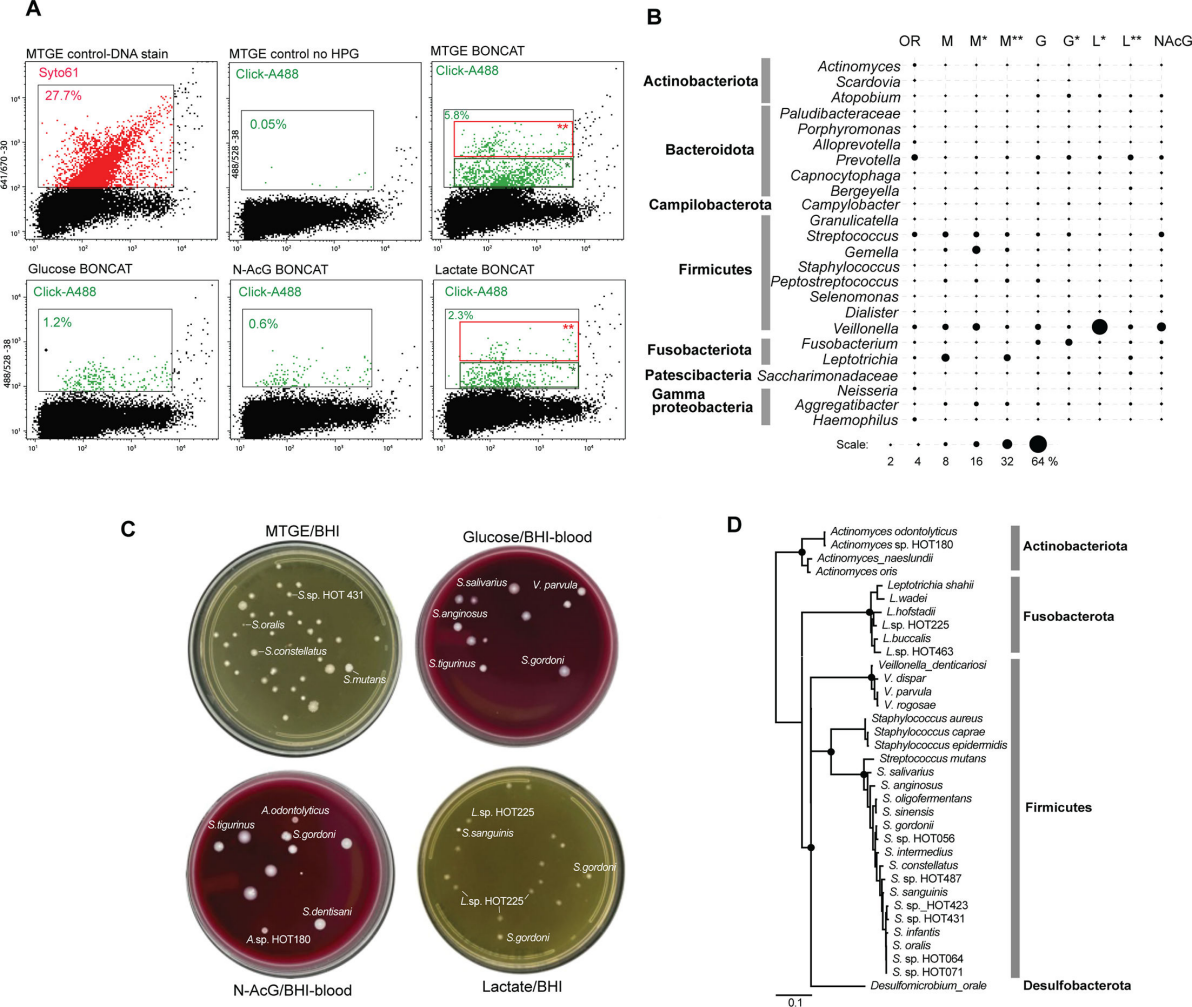
Oral microbiota BONCAT-Live. (**A**) Flow cytometry of human oral microbiota (saliva/supragingival biofilm sample) labeled after BONCAT incubation in MTGE medium or in basal medium supplemented with glucose, N-acetylglucosamine, or lactate. The different gates used for sorting and particle/cell frequency are indicated. (**B**) Microbial taxa relative abundance (top genera) in original sample (OR) and BONCAT-positive sorted cell populations based on gating shown in panel **A**. (**C**) Bacterial isolates following BONCAT-positive single-cell sorting on brain heart infusion (BHI) nutrient agar. The identity of representative colonies is indicated. (**D**) SSU rRNA phylogeny of isolated bacterial strains based on closest human oral relatives.

Collectively, the oral microbiota has complex nutritional requirements that may compete with the uptake/incorporation of HPG by some of its members. Therefore, to evaluate the breadth of BONCAT coverage under our experimental conditions, we first performed incubations in a non-selective, nutrient-rich medium (medium of tryptone, glucose, and yeast extract[MTGE]) that supports the growth of many oral fastidious bacteria. Out of the 69 bacterial genera detected in the starting oral sample by amplicon sequencing, 52 were present in the BONCAT-labeled sorted population (Fig. 6; Table S2). Notable taxa that increased in relative abundance, based on both DNA staining and BONCAT, included *Gemella*, *Peptostreptococcus*, *Veillonella*, *Leptotrichia*, and *Aggregatibacter*, while some displayed lower levels (*Actinomyces, Prevotella*). In particular, *Leptotrichia* (Fusobacterota) and *Streptococcus* (Firmicutes) had the strongest fluorescence signal, suggesting high translational activity.

We then used a medium base with a lower nutrient content (artificial saliva) to determine how individual bacteria respond to the addition of selected growth substrates. Glucose and other sugars impact the metabolism of a variety of oral bacteria and, through acidogenic effects and community changes in the dental biofilm, are linked to caries (61). Based on the BONCAT signal, the most active taxa upon being provided glucose were *Atopobium* (Actinobacteriota), *Prevotella* (Bacteroidota), *Streptococcus*, and *Fusobacterium*. Lactate, a product of carbohydrate metabolism, is also utilized by many oral species. The transition from sugars to lactate and organic acid utilization has been well documented, as shown by functional genomics (62). Lactate-containing media led to the highest activity by *Prevotella*, *Veillonella*, and *Leptotrichia*, while *Streptococcus* subsided (Fig. 6; Table S3). The third substrate that we tested, N-acetyl glucosamine (N-AcGlc), is an important component of the extracellular matrix, mucins, and bacterial cell walls. While some species recycle such amino sugars as peptidoglycan building blocks, nutritional utilization supports a diverse community through spatial, physiological niche partitioning, and competition, e.g., between species of *Streptococcus* (63–66). In our incubations, N-AcGlc had the smallest effect in inducing high translational activity (BONCAT signal), presumably due to a slower rate of uptake and a more restricted utilization as compared to the other tested nutrients. Nevertheless, *Streptococcus* and *Veillonella* exhibited strong signals, corroborating their known metabolic and physical interaction within the community (62, 66, 67).

Single cells from the BONCAT-positive populations identified by flow cytometry were deposited on non-selective nutrient agar and incubated anoxically until colonies formed. The near full-length 16S rRNA gene amplicons enabled assignments of those isolates to known human oral bacteria species/strains. Across all tested nutrients described above, we isolated and assigned strains to over 30 human oral bacteria (Fig. 6B). Expectedly, most colonies represented a variety of strains of *Streptococcus*, *Leptotrichia*, and *Veilonella*, which were identified as highly abundant and active based on BONCAT population-level amplicon data. We also isolated representatives of *Actinomyces* and *Staphylococcus*, which were in the minority of BONCAT responders. On the other hand, we did not retrieve some other relatively abundant BONCAT-positive bacteria, including *Prevotella*, *Gemella*, and *Fusobacterium*. This may have been due to inhibition caused by BONCAT/FACS sorting or non-permissive culture conditions for those strains, which we did not attempt to distinguish and address here.

## DISCUSSION

Traditional and high-throughput cultivation strategies have expanded the diversity of cultured organisms. However, most approaches do not provide direct insights into the range of physiological aptitudes of those isolates. Cultivation-independent approaches have been designed to ascertain physiological potentials *in situ*, including SIP and various meta-omics (12, 13, 68–70). BONCAT and other click chemistry-enabled cellular labeling techniques complement sequence-based interrogations of active microbes in their environmental settings. Combined with advanced spectrometry imaging techniques (nano-secondary ion mass spectrometry and Raman), isotope probing and BONCAT enable cell-level resolution assignment of metabolic and biogeochemical processes (71–73). Ideally, cells labeled based on their physiological properties should not only be identified destructively but also viably retrieved for cultivation, for further characterization, and for downstream applications.

BONCAT-Live directly enables isolation and cultivation of microbial strains that are physiologically active or activated under specific conditions on environmentally relevant time frames. Because the initial “growth” (protein synthesis that enables cell labeling) can occur in the native sample, the cells are exposed to some of their naturally occurring physical, chemical, and biological factors. In two of the test cases we analyzed, rhizosphere and permafrost, samples were handled and incubated under conditions to maintain their native architecture as much as possible, so that microbial communities are primarily responding to the testing variables. Because the oral microbiota could not be studied *in vivo*, we tested responses to chemical cues under laboratory conditions permissive to some of the community members. Most of the relatively abundant BONCAT-positive taxa were retrieved as isolates, and Sanger sequencing of 16S rRNA genes resolved higher strain diversity than is attainable with typical short-amplicon data (e.g., the permafrost *Cryobacterium* and *Arthrobacter*, the rhizosphere *Pseudomonas*, and the oral *Streptococcus* and *Leptotrichia*; Fig. 4 to 6). Applying multiple sorting gates (Fig. 5 and 6) also led to isolating some low-abundance positive taxa, half of the diversity of isolates being present at less than 1% abundance in the BONCAT-positive population. Therefore, while not a quantitative assay, single-cell isolation and cultivation by BONCAT-Live can provide greater confidence in the microbial activity of individual taxa under specific conditions than can sequences retrieved by cultivation-independent amplicon or metagenome sequencing. Follow-up characterization of such strains would be necessary to test their physiological properties and how they relate to the BONCAT experiment variables or the biological/environmental questions being tested. For example, when investigating metabolites/compounds that can act as inhibitors, physiologically active microbes may be resistant to their effects but utilize other existing nutrients or use them as nutrients, either independently or in association with other members of the community through cross-feeding. Salicin is one such example we tested in the rhizosphere. The rapid, sharp increase in *Pseudomonas* and the *Micrococcaceae* actinobacteria (Fig. 3), both metabolically versatile taxa, suggests they may have utilized salicin directly. A variety of other taxa proliferated as well as compared to the native rhizosphere incubation, leading to a nearly fourfold increase in the frequency of BONCAT-positive cells. Salicin utilization assays would be required to identify which of those isolates can uptake it and which use the salicyl alcohol resulting from salicin hydrolysis by other strains (45). The nature of the substrate and the selected time frame are therefore important considerations in designing the BONCAT assay to probe the nutritional dynamics and to separate primary users from secondary cross-feeders. Because taxa retrieval in BONCAT-Live is not quantitative, time course amplicon data of BONCAT-positive sorted populations could indicate such microbial succession and guide which isolates may correspond to which category, subject to physiological testing. The dynamics of the community and succession of various members can be approached by BONCAT and BONCAT-Live under a variety of settings and time frames, including *in situ* (21). An advantage of directly isolating strains responding to experimental variables by BONCAT-Live is that it directly validates the translational activity of strains under the tested conditions (including, e.g., the reaction to antimicrobials and specific nutrients).

Each of the technical steps of BONCAT-Live can affect the viability of individual taxa differently, which constitutes a limitation of the approach. Copper was, in our testing, the most problematic and, while reducing its concentration and the exposure to oxygen radicals improved viability of test bacteria and enabled isolation of a wide variety of other taxa, it is conceivable some microbes may not tolerate. While Cu-independent click chemistry labeling is a complementary option to be further explored, it introduces additional chemistries and incubation conditions that may also be detrimental to some microbes. Because BONCAT-Live provides the initial single-cell isolation step from its native community, it is exposed to some of the same shortfalls of standard isolation by plating, namely, effective recovery of viable organisms from their environmental matrix, media selection, and incubation conditions, which would have to be explored and optimized, depending on samples and target organisms. For example, selected single cells could be deposited on plates or in liquid culture that harbor helper organisms or grown as mini-communities by co-sorting from the BONCAT-positive population. Magnetic bead-based enrichments, demonstrated in our test experiments, could be an alternative to retrieve and culture populations of microbes that are co-dependent, refractory to FACS, or highly sensitive to oxygen when all steps are performed in an anoxic chamber.

By broadly targeting translationally active bacteria, BONCAT-Live is taxon-agnostic. Taxonomic identification of bacteria and archaea has been previously linked to BONCAT using FISH with specific probes following fixation (23, 71). Taxon-specific cell surface antibodies may also be used to isolate specific taxa by BONCAT-Live in combination with targeted reverse genomics (74). Using standard FACS equipment, BONCAT-Live can be readily implemented as a direct approach to isolate and culture metabolically active single cells from environmental samples, complementing culture-independent and traditional cultivation strategies to characterize communities and processes. A distinct approach that also enables isolation and cultivation of active cells, Raman-activated cell sorting can target various metabolic activities within the cells but requires custom instrumentation and has lower analytical throughput than fluorescence-based cell sorting (75). While we only isolated bacterial strains, BONCAT has been shown to be compatible with labeling of archaea, eukaryotes, and viruses as well (17, 21, 23, 71, 76). Therefore, the approach may be applicable to broader taxa and physiotypes than those accessed here. BONCAT-Live does not only enable selecting a physiological/metabolic characteristic as the driver for isolation but also, combined with FACS fractionation, provides access to low-abundance taxa, often inaccessible by direct plating. The bacterial strains responding to specific stimuli that we isolated here from various environments will serve for further characterization, individually and in synthetic communities.

## MATERIALS AND METHODS

### Environmental samples

#### Arctic samples

In March 2021, eight boreholes were drilled at Ny-Ålesund, Svalbard, Norway, at the Bayelva long-term permafrost monitoring site (78.92094° N, 11.83334° E) (56). For this study, we used a core collected at borehole 7, where the permafrost underlies the active layer at about 1.5 m and below. The soil is silty clay to sandy silt and is characterized as highly lithic and low in nutrients (60). The cores were shipped frozen and were stored at −20°C.

#### Rhizosphere, soil, and sediment samples

Near-surface (2–10 inches deep) clusters of fine roots (<3 mm diameter) and associated soil were collected with a trowel from 4- to 5-year-old *Populus deltoides* trees at an experimental site on the ORNL reservation in Oak Ridge, TN, USA, during active growth season (April-October). The soil and roots were transported in closed sterile bags to prevent drying in an insulated box to the laboratory and used within 3 days of collection.

#### Oral samples

Supragingival and gum line plaque was self-collected from premolars-molars on each mouth side using two OMR-110 swabs (DNA Genotek, Ottawa, Canada). The swab tips were immediately placed in a vial of liquid dental transport (LDT) medium (Anaerobe Systems, Morgan Hill, CA, USA) and processed within 2 hours.

Aliquots of all collected samples were stored frozen at −80°C for baseline microbial community characterization.

### BONCAT incubations

#### Cultured bacterial strains

For control tests and optimization experiments, we used previously isolated *Populus* rhizosphere soil strains (*Pseudomonas* sp. GM41, *Terriglobus* sp. ORNL, *Flavobacterium* sp. CF108, *Bacillus* sp. OV166, *Rhizobium* sp. CF142, *Paraburkholderia* sp. BT03, *Roseimicrobium* sp. ORNL1, *Sphingomonas* sp., and *Dyella* sp.) (31, 76, 77), Svalbard permafrost *Pseudomonas* sp. (59), as well as two sulfate reducers, *Desulfovibrio desulfuricans* DSM 642 and *Desulfohalobium retbaense* DSM 5692. The soil strains were cultured in a low methionine version of R2A (based on DSMZ medium 830 formulation) in which we substituted the peptone and casamino acids with 1 g/L of a synthetic, with equal mix of all other 19 amino acids (referred to as defined R2A or R2A*) at 25°C. The two sulfate reducers were grown anoxically in DSMZ media 63 and 499, respectively, at 30°C. Each strain was inoculated in 5 mL media with or without 50 µM HPG (Vector Labs, Newark, CA, USA) and incubated for 48 hours. For killed incorporation controls, cultures fixed with 3% paraformaldehyde were supplemented with HPG and incubated similarly. At the end of incubations, cells were collected by centrifugation (10 min at 8,000 × *g*), washed three times in phosphate-buffered saline (PBS), and resuspended in PBS. Aliquots were stored in PBS containing 10% glycerol and 1% trehalose at −80°C or used immediately for click-chemistry labeling.

#### Rhizosphere incubations

Fragmented roots and associated rhizoplane soil (5 g) were spread on the surface of Petri plates and soaked with 10 mL 0.25× Hoagland’s No. 2 hydroponic mineral nutrient solution (Sigma-Aldrich, St. Louis, MO, USA) at 25°C for 18 hours. An additional 10 mL supplemented with 100 µM HPG was added, or supplement also containing 40 mM salicin (Sigma-Aldrich), or (40 mM) potassium malate (Sigma-Aldrich). Background control incubations contained no HPG or paraformaldehyde (3%). After gentle mixing, the plates were incubated for an additional 24 hours at 25°C. The roots, soil slurry, and incubation solution were transferred into 50 mL Falcon tubes, supplemented with 10 mL PBS, vortexed for 1 minute, and centrifuged at 1,000 × *g* for 2 minutes. The supernatant was then filtered through a 50 micron CellTrics strainer (Sysmex, Lincolnshire, IL, USA) and spun at 8,000 × *g* for 10 minutes to pellet the microbial cells. The pellet was washed three times as above. Sample aliquots were used immediately for click-chemistry labeling and FACS, and others preserved at −80°C in PBS-glycerol-trehalose.

#### Arctic soil incubations

Frozen soil samples (~15 g) from active layer and permafrost horizons of the cores were distributed in 50 mL vented, 0.22 µm filter-capped Falcon tubes on ice. Seven milliliters of ice-cold sterile 50 nM HPG solution in water was added to penetrate the soil. Control incubation samples received only water or HPG in 3% paraformaldehyde (killed control). To simulate permafrost locations with higher organic matter content, a set of incubations was supplemented with 10% R2A*. Incubations were performed at 4°C (2–6 weeks) or at −20°C (12 weeks). Following incubation, the samples were processed as described for the rhizosphere.

#### Oral microbiome incubations

To maintain the viability of anaerobic bacteria, all oral sample processing was performed under anoxic conditions in a COY anoxic chamber with an atmosphere of 85% N_2_, 10% CO_2_ and 5% H_2_. The vial with oral swabs in LDT was transferred into the chamber and vortexed for 1 minute. The liquid was passed through a 10 µm CellTrics strainer into Eppendorf tubes, centrifuged for 10 minutes at 8,000 × *g*, and the pellet was washed and resuspended in 2 mL PBS. Aliquots (100 µL) were added into 1 mL BONCAT incubation media with 100 µM HPG that included rich, non-selective MTGE medium (Anaerobe Systems) or chemically defined artificial saliva medium (defined medium mucin [DMM]) (78) supplemented with either 10 mM glucose, 10 mM sodium lactate, or 10 mM N-acetyl glucosamine. As controls, samples were inoculated in MTGE medium with no HPG or with 3% paraformaldehyde. After incubation for 2 hours at 37°C, the samples were centrifuged, washed three times with PBS, and immediately used for click chemistry labeling.

### Click chemistry biotinylation for imaging and magnetic enrichment

Cultured test bacteria grown in the presence of HPG and their corresponding negative controls (killed and non-HPG) were used individually for i- suspension labeling experiments guided by a published protocol (79). Freshly prepared aminoguanidine hydrochloride (50 µL, 100 mM in PBS) and sodium ascorbate (50 µL, 100 mM in PBS) were added to a 0.9 mL cell suspension (OD_600_ = 1.0) in PBS in a 1.5 mL Eppendorf tube and mixed by inversion. A separately prepared mix containing CuSO_4_ (5 µL, 20 mM in water), Tris(1-hydroxypropyl-1H-1,2,3-triazol-4-yl)methyl]amine (THPTA, Click Chemistry Tools) (10 µL, 50 mM in water), and biotin picolyl azide (Click Chemistry Tools) (5 µL, 2 mM in dimethyl sulfoxide), pre-reacted for 3 minutes, was pipetted in the tube, and the tube inverted once. Following a 30 minute incubation in the dark, cells were pelleted, washed three times in PBS, and resuspended in 1 mL PBS. For imaging, 50 µL aliquots were mixed with a 50 µL PBS solution containing 1% biotin-free bovine serum albumin (BSA) (Fraction 5, Sigma-Aldrich), 0.02% Pluronic F127 surfactant (Sigma-Aldrich), and 5 µg/mL streptavidin-Alexa Fluor 488 (green) or Alexa Fluor 546 (red-orange) conjugate (Thermo Fisher Scientific) and 1 µg/mL Hoechst 33258 (DNA blue stain for live cells, Molecular Probes), incubated in the dark for 30 minutes, washed three times with PBS-0.02% Pluronic F127 and visualized/photographed under epifluorescence on a Zeiss AxioImager M2 microscope.

For the magnetic enrichment experiments (performed at different times), we obtained enriched microbial fractions from 10 g of environmental samples (rhizosphere soil from the *Populus* plantation, garden soil, and water pond surface sediment) using centrifugation on Histodenz, as we described previously (80). Approximately 5 × 10^8^ enriched bacteria (based on an estimation of 1OD_600_ = 10^9^ cells) was mixed with 10^8^ of selected individual biotinylated strains or controls (no-HPG and PFA-killed) and adjusted to 1 mL in PBS. An aliquot (50 µL) was saved for community diversity characterization, and the rest was mixed with 1 mL PBS containing 1 mg/mL biotin-free BSA, 0.02% Pluronic F127, and 2 mM EDTA (PBS*). We then added 200 µL Streptavidin MicroBeads (Miltenyi Biotech, Gaithersburg, MD, USA) and incubated the suspension at 4°C for 2 hours on a rotator. The cell-bead suspension was then pelleted by centrifugation (8,000 × *g*, 10 minutes at 4°C), washed twice with 1 mL PBS* and resuspended in 0.5 mL PBS*. An LS MACS column and separator (Miltenyi Biotech) was then used, following the manufacturer’s protocol, to purify the magnetically labeled biotinylated cells. The eluted cell fraction, along with aliquots of original community and the column-applied mixes, was used for DNA extraction with the ZymoBIOMICS DNA Microprep Kit (Zymo Research, Irvine, CA, USA).

### Viability tests and click chemistry labeling for BONCAT-Live

To test the impact of various chemical compounds on viability, we used the soil rhizosphere strain *Pseudomonas* sp. GM41, the output being colony formation after single-cell sorting on nutrient agar. HPG or AHA (100 µM) was tested in 48 hours of incubation in R2A*. Each of the major chemicals used in click-chemistry reactions was individually tested at starting concentrations recommended for click labeling reactions (79) (sodium ascorbate 5 mM, aminoguanidine hydrochloride 5 mM, 2-chloroacetamide 10 mM, CuSO_4_ 100 µM, THPTA 500 µM, and AZDye488 picolyl azide 1 µM) by incubation with cells suspended in PBS at room temperature for 30 minutes (37°C for chloroacetamide), followed by cell washing in PBS, DNA labeling with Syto61, and FACS. Parallel tests were conducted with the complete click-labeling reaction protocol, as described above and in reference 79. The only major negative impact was observed for 2-chloroacetamide (~80% inactivation) and CuSO_4_ at 50–100 μM (>50% inactivation). Consequently, we did not further use AHA and the strain-promoted (Cu-free) click-chemistry protocol, and we focused on improving the viability with Cu-catalyzed reactions by repeating the tests with reduced copper concentrations. Three combined modifications to the standard protocol restored viability (>80%) while maintaining effective fluorescent labeling with 2 µM AZDye488 Picolyl Azide. First, CuSO_4_ concentration was reduced to 5 µM. Second, we substituted THTPA with BTTAA (Click Chemistry Tools), which has been claimed to have lower cytotoxicity (81). Although THTPA was not inhibitory to *Pseudomonas*, we aimed at reducing potential negative effects on other microbes in the environmental samples. Third, we added to the cell suspension (prior to introduction of reaction chemicals), 1 mM sodium pyruvate (Sigma-Aldrich) and 100 U/µL catalase (bovine liver, Sigma-Aldrich), reactive oxygen scavengers reported to improve microbial viability (38, 40, 82). We did not individually test their effects. These conditions were then applied to labeling of microbiota samples following BONCAT incubations. For click reactions with oxygen-sensitive oral microbiota samples, we prepared all fresh stock solutions and degassed buffer under nitrogen gas bubbling with long needles inserted in the Falcon tubes followed by capping and transfer to an anoxic chamber.

For microbiota samples that we used in BONCAT-Live sorting experiments, samples were mostly labeled fresh, immediately after BONCAT incubations. We also labeled and sorted for cultivation soil samples that were cryopreserved (primarily from permafrost incubations). While it is likely not all microbes will survive freezing, we did not collect sufficient isolates to distinguish differences. Because immediate isolation is not always feasible after BONCAT, click labeling and FACS are therefore applicable to preserved samples as well.

### FACS and cultivation

Prior to flow cytometry analyses, pure cultures used for viability testing and microbiota samples were counterstained for DNA with 0.5 µM Syto61 red fluorescent dye (Thermo Fisher Scientific). For flow cytometry and cell sorting, we used a Cytopeia/BD Influx Model 208S (BD, San Jose, CA, USA). The sheath fluid was 0.5× PBS (filtered through a 0.1 µm filter), pressurized at 18 psi. For cytometry analyses and sorting, we used a 70 µm nozzle and the 488 nm laser for forward scatter-side scatter (FSC-SSC) and non-fluorescent sorting and the 488 and 641 nm lasers for fluorescent cell sorting. Flow sorting under these conditions does not have a major impact on viability, as we have consistently recovered >80 colonies out of 100 sorted cells of various cultured bacteria with or without including a DNA staining dye. For comparison, we have obtained >40% cultivability after random single-cell sorting of samples from various environments (Fig. S2). Because some particles may not be live cells, the number of colonies could underestimate sorting-linked viability estimations. Gating parameters were selected based on FSC-SSC and red/green fluorescence levels for each experiment and sample to include DNA staining and, when click labeled with AZDye488, BONCAT surface staining. For sorting pools of cells from selected gated populations, 5,000–20,000 cells (corresponding to ~5 to 20 μL volume in our flow sorter) were deposited per well containing 3 mL sterile, UV-irradiated TE (83) in a 96-well plate (10 mM Tris-HCl, pH 8, and 1 mM EDTA). In our workflow, a minimum of 5,000 sorted events/bacterial cells (approximately 5 μL) were determined necessary for reliable generation of rRNA amplicon libraries for sequencing. The pools of cells were stored at −80°C after sealing with sterile foil.

To sort single cells for cultivation, we used standard round or rectangular culture plates with selected nutrient media agar for the different microbiota samples. The sorting chamber was sterilized by UV with the instrument germicidal lamp for 15 minutes. Deposition of 100 cells per plate was performed with sorting set to single-particle pure mode. We typically sorted three to four plates per sample or gate. After sorting, plates were incubated at 4°C (permafrost, 7+ days), 25°C (rhizosphere and permafrost, 24–72 hours). For experiments with oral microbiota samples, we prepared the instrument for anoxic sorting, as previously described (27). Briefly, the sheath fluid was heated to 95°C in the pressure vessel and cooled under nitrogen. The flow sorter gas lines were switched to nitrogen. The oral samples in sorting tubes, prepared and capped in the anoxic chamber, were inserted in the instrument sample port and pressurized with nitrogen gas. Following single-cell deposition of oral bacteria on anoxic culture media, the plates were immediately transferred into acrylic boxes with oxygen-absorbing AnaeroPacks (Mitsubishi Gas Company) and incubated at 37°C (24–72 hours).

### Molecular microbial characterization

DNA was extracted from aliquots of starting environmental samples with the ZymoBIOMICS DNA Microprep Kit. Sorted pools of cells were subject to lysis by three cycles of snap freeze-heat between a metal 96-well block on dry ice followed by a PCR block at 95°C (3 minutes each). We have determined that such a freeze-heat treatment is an effective lysis method for the recovery of most microbial taxa as compared to DNA extraction, which is not feasible for minute samples (see the supplemental material). DNA and cell lysates were then used for microbial community characterization by SSU rRNA gene amplification (V4 region) using Illumina-adapted, barcoded universal primers 515F and 806R (detailed in reference 84), with the Quick-16S NGS Library Prep Kit (Zymo Research), according to the manufacturer’s protocol. For the first step target V4 amplification, we sometimes increased the reaction volume to 40 µL and used up to 16 µL of the sorted pools of lysed cells obtained by FACS (theoretical 1,000 cells per μL lysate) to yield sufficient amplification. The bidirectional amplicon sequencing was performed on a MiSeq instrument (Illumina, San Diego, CA, USA) using the v.2 500 cycle kit, according to the manufacturer’s instructions. At least 15,000 paired reads were obtained per sample (read counts listed in Sequence Read Archive metadata). Demultiplexed raw sequence reads were imported into Qiime2 (85), denoised and paired, and chimeras were removed with the dada plugin and analyzed for taxonomic composition and relative abundance using standard workflow as described in references 77, 86, and 87. Negative controls yielded primarily unassigned reads or mapped to known PCR reagent contaminants (*Escherichia coli*), and those were removed from all samples. Amplicon sequence variants representing less than 100 sequences across all samples were also removed from final comparisons, as standard practice in the field. Bubble plots representing the top 20–30 relatively abundant taxa were generated using a Perl script (88).

To identify the microbial isolates, near full-length SSU rRNA gene amplification was performed by colony PCR with bacterial universal primers (27F-1492R), followed by Sanger sequencing (Eurofins Genomics, Louisville, KY, USA). For each sample/treatment/sorting gate, we selected visually distinct colonies (size, appearance, and color) to represent the observed diversity on plates. The sequences were imported in Geneious Prime v.2021 (89), trimmed based on quality, and compared to relatives (cultured and uncultured) in GenBank using BLASTn. For the oral isolates, comparisons were done using the Human Oral Microbiome Database (https://www.homd.org/) (90). Sequences of our isolates and their closest relatives were aligned in Geneious using MUSCLE (91) and used for phylogenetic tree reconstruction with FastTree (92). The figures were composed and labeled in Adobe Illustrator. Representative clones spanning the recovered taxonomic diversity were propagated in liquid media and preserved at −80°C in glycerol-containing media.

## Data Availability

Bacterial isolates are available upon request. The Illumina amplicon sequences have been deposited at the NCBI Sequence Read Archive under accession numbers SRR34335762–SRR34335793, under BioProject number PRJNA1285074. The 16S rRNA Sanger sequence data for the isolates are provided as a supplemental fasta file.

## SUPPLEMENTAL MATERIAL

Fig. S1 mbio.02389-25-s0001.pdf Cell surface BONCAT.

Fig. S2 mbio.02389-25-s0002.pdf Viability tests for flow sorted bacteria.

Fig. S3 mbio.02389-25-s0003.pdf Flow cytometric profiles.

Sequence data mbio.02389-25-s0004.txt Isolate Sanger sequences.

Supplemental tables mbio.02389-25-s0005.xlsx Tables S1 to S3.

ASM does not own the copyrights to Supplemental Material that may be linked to, or accessed through, an article. The authors have granted ASM a non-exclusive, world-wide license to publish the Supplemental Material files. Please contact the corresponding author directly for reuse.
