# Supplementary material for: BONCAT-Live for isolation and cultivation of active environmental bacteria
Source: mBio. 2025 Sep 22;16(11):e02389-25. doi: 10.1128/mbio.02389-25 (PMC12607769; doi:10.1128/mbio.02389-25)
Supplement: Fig. S1 — Cell surface BONCAT. [file mbio.02389-25-s0001.pdf]

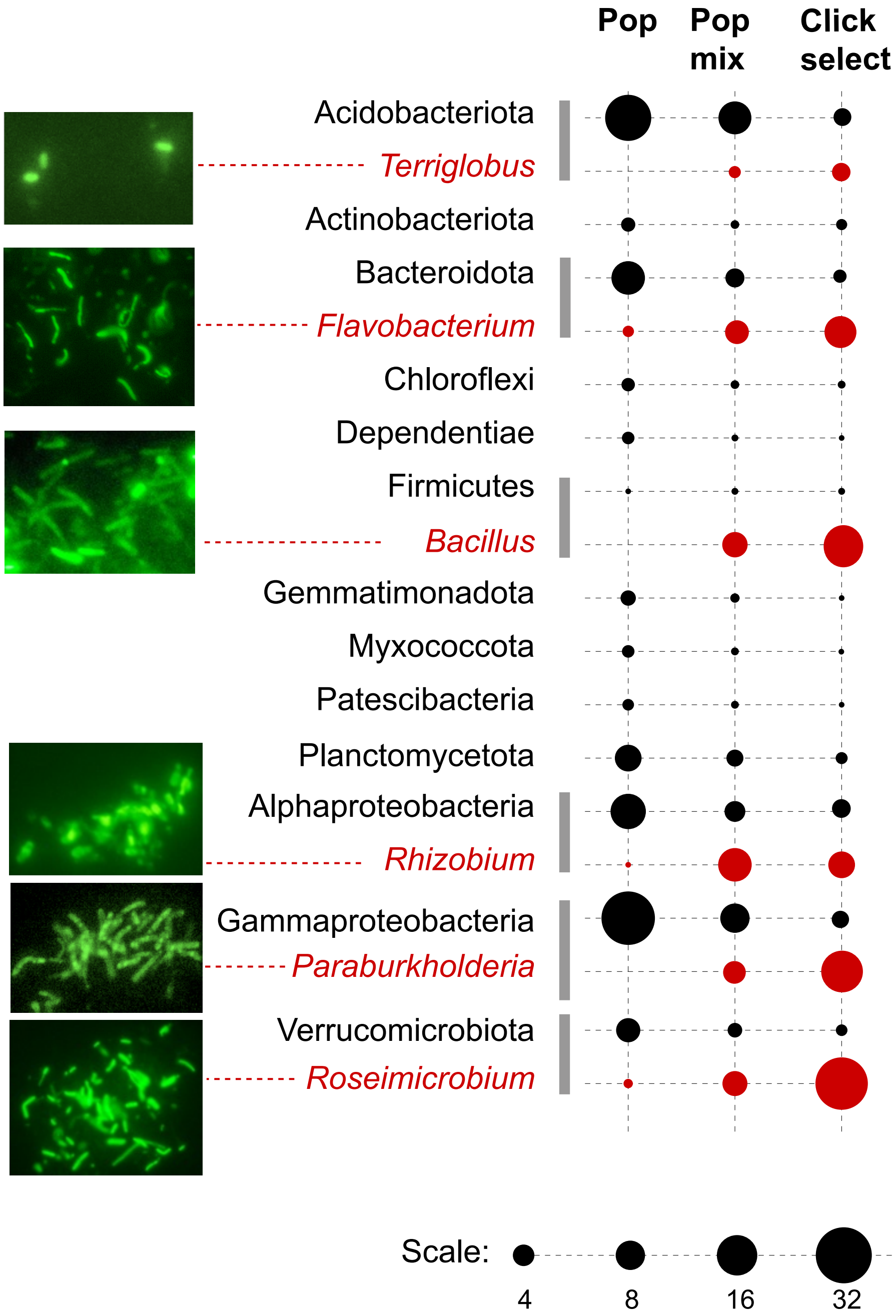

Figure S1. Cell surface BONCAT labeling and selection for rhizosphere bacteria. Strains representing six bacterial genera (red) were labeled with biotin after BONCAT and imaged after surface staining with Streptavidin-A488 or magnetically enriched after seeding in a soil microbiota sample. Images of non-fluorescent negative controls are not shwon. The bubble plot indicates taxon relative abundance based on SSU rRNA V4 amplicons comparing the soil sample before and after seeding with the magnetically enriched eluate.
