# Supplementary material for: BONCAT-Live for isolation and cultivation of active environmental bacteria
Source: mBio. 2025 Sep 22;16(11):e02389-25. doi: 10.1128/mbio.02389-25 (PMC12607769; doi:10.1128/mbio.02389-25)
Supplement: Fig. S2 — Viability tests for flow sorted bacteria. [file mbio.02389-25-s0002.pdf]

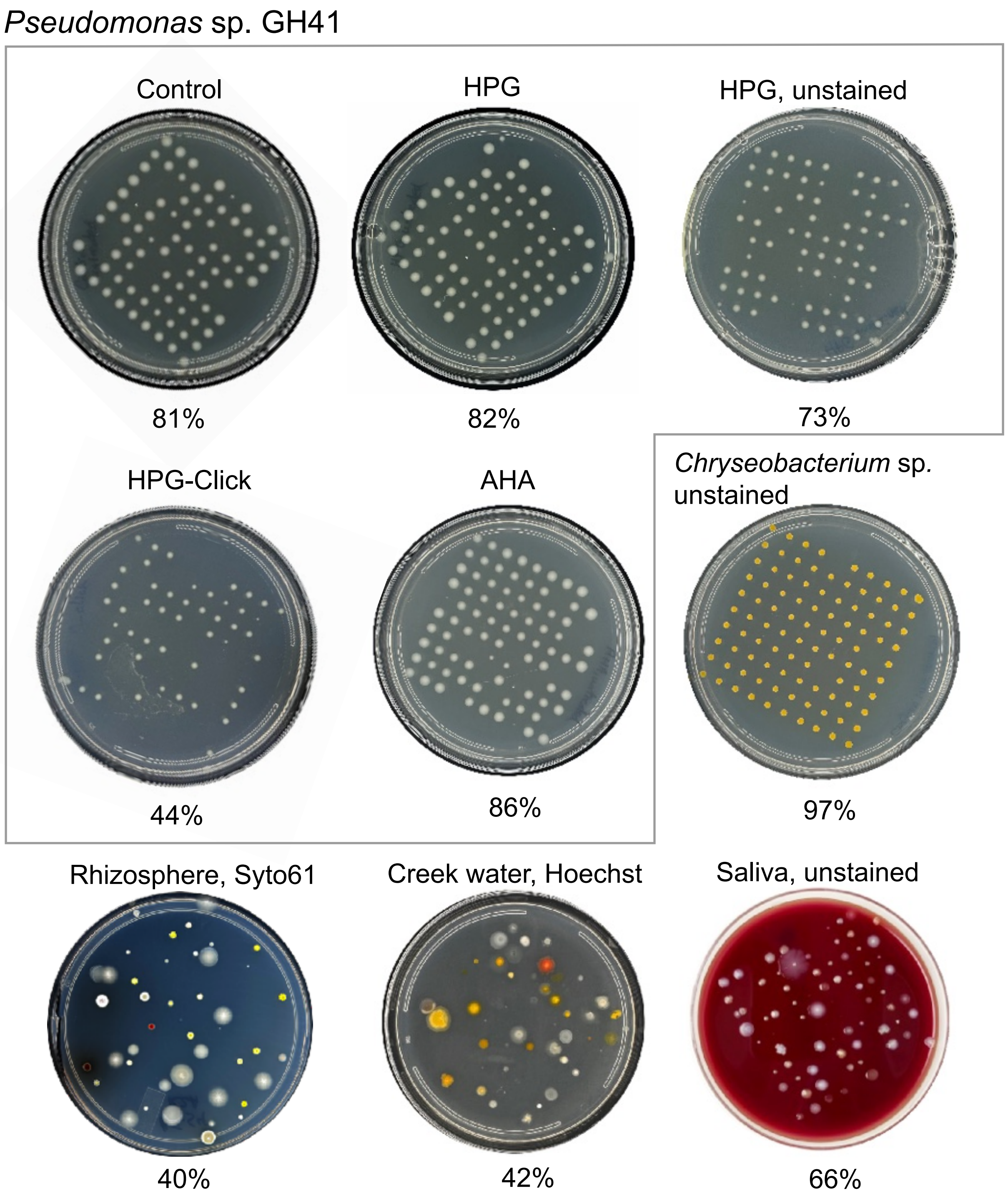

Figure S2. Viability tests for flow sorted bacteria. On each test, 100 fluorescent cells/particles were individually deposited on R2A agar plate, followed by incubation (2 days at 25C). Control, untreated cells grown in R2A*; HPG and AHA, cells grown in R2A* supplemented with HPG or AHA, respectively; HPG-Click, cells grown in R2A* supplemented with HPG that were click-labeled using copper chemistry protocol; HPG unstained, same as HPG but not stained for DNA, sorted by scatter. For comparison, untreated *Chryseobacterium* sp. and three environmental samples were also sorted (*Populus* rhizosphere and fresh water creek, on R2A, and human saliva, on BHI-blood). The percentages indicate number of colonies (viability/cultivability).
