# Supplementary material for: BONCAT-Live for isolation and cultivation of active environmental bacteria
Source: mBio. 2025 Sep 22;16(11):e02389-25. doi: 10.1128/mbio.02389-25 (PMC12607769; doi:10.1128/mbio.02389-25)
Supplement: Fig. S3 — Flow cytometric profiles. [file mbio.02389-25-s0003.pdf]

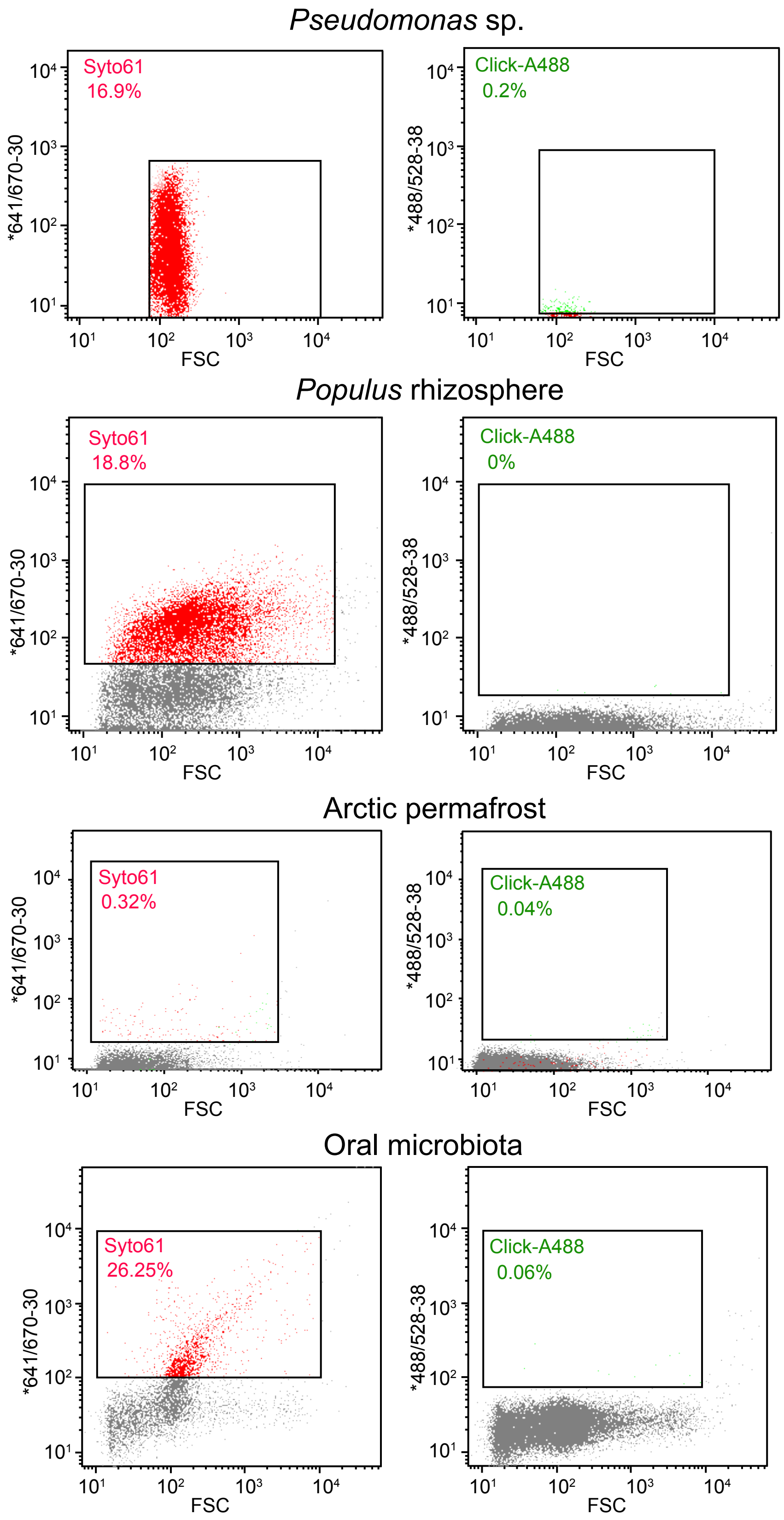

Figure S3. Flow cytometric profiles of PFA-killed HPG samples controls, stained for DNA with Syto61 and labeled by click-chemistry with A488.
